# Analysis of Selected Soil Properties in Relation to Soil and Water Conservation Practices in Sibiya Arera, Soro District, South Central Ethiopia

**DOI:** 10.1155/2023/1763367

**Published:** 2023-02-28

**Authors:** Belayneh Bufebo, Getachew Beyene, Temesgen Addise

**Affiliations:** Department of Natural Resource Management, Wachemo University, P.O. Box 667, Hosanna, Ethiopia

## Abstract

Soil erosion by water is a severe and continuous ecological problem in the south central highlands of Ethiopia. Limited use of soil and water conservation technologies by farmers is one of the major causes that have resulted in accelerated soil erosion. Within this context, significant attention has been given to soil and water conservation practices. This study was conducted to investigate the effects of soil and water conservation practices on soil physicochemical properties after being practiced continuously for up to 10 years. The physicochemical properties of soil of landscape with physical soil and water conservation structures without biological conservation measures and physical soil and water conservation structures combined with biological conservation measures were compared with soil of landscape without soil and water conservation practices. The result of analysis disclosed that soil and water conservation interventions (both with biological and without biological measures) significantly increased the soil pH, soil organic carbon, total nitrogen, and available phosphorus content than the soil of landscape without soil and water conservation practices. The results of the analysis also showed that the mean value of cation exchange capacity and exchangeable bases (K^+^, Na^+^, Ca_2_^+^, and Mg_2_^+^) of the soil under nonconserved farm field was significantly lower as compared to the soil of adequately managed farm fields. The findings of this study clarified that there was significant variation in soil properties. This variation could be due to uneven transport of soil particles by runoff. Therefore, soil conservation structures supported with biological measures improves the soil's physicochemical properties.

## 1. Introduction

The soils constitute one of the world's most important natural resources. Soil plays a crucial role in biochemical and geochemical cycling, partitioning of water, storage and release, buffering, and energy partitioning, which are essential for supporting ecosystems [1]. Soil, as a major component of land resources, from which many ecosystem services essential to humans and the environment is obtained [2].

Soil is one of the natural capitals that provides long-term economic, production, and environmental services and plays a major role in global climate processes through regulation of carbon dioxide, nitrous oxide, and methane emissions; however, agricultural management practices can largely influence the quality of the soil [3]. Because of humans' activity for long-term economic production soil is becoming extremely susceptible to various forms of depletions, such as soil erosion, soil fertility decline, and associated changes in soil physicochemical properties [4].

The great efforts has been made to address the problem of soil erosion in Ethiopia since 1970s [5] coming after the occurrence of famine and drought. Since then, the government of Ethiopia has given considerable attention on soil and water conservation technologies for rehabilitation of land resources. A large number of conservation, rehabilitation, and afforestation campaigns were undertaken through Food-For-Work programs. Nevertheless, the efforts have not been happening in many places and some farmers are still reluctant showing no willingness to practice soil and water conservation measures in their farm fields [6].

Agriculture is the backbone of the Ethiopian economy accounting more than 80% of total employment, 84% of national export, and 50% of gross domestic product (GDP). However, currently, there is an increasing concern that soil erosion as the result of improper land resources management extremely limits sustainability of agriculture in Ethiopia [7]. Activities like deforestation, overgrazing, and intensive cultivation without conservation measures, caused severe soil erosion, which diminished agricultural production and affected food security. The estimated soil losses in Ethiopia due to erosion at the rate of 130 tones/ha for cultivated fields and 35 tons/ha average for all land use classes in the highland areas of the country [8]. The government of Ethiopia by mobilizing local community has been implemented soil and water conservation practices since the last four decades through watershed management approach [9]. The most commonly constructed physical soil and water conservation structures to control soil erosion are fanya juu. Fanya juu terraces are made by digging a trench along the contour and throwing the soil uphill to form an embankment. The embankments are stabilized with fodder grasses and in between cultivated portions. Over time, the fanya juu develop into bench terraces.

Soro district is located in south central Ethiopia where soil erosion has been a major problem resulting in soil fertility depletion. In reaction to extreme problem of soil degradation, soil and water conservation practices have been implemented in different areas of the district. Among areas, Sibiya Arera is the one on which soil and water conservation practices have been constructed. Besides, to stabilize physical soil and water conservation structures (fanya juu) desho grass (*Pennisetum pedicellatum*) has been planted. However, landscape with soil and water conservation practices in relation to soil physicochemical property is not studied so far in the study area. As the result; such effects of soil and water conservation practices on soil physicochemical properties is poorly understood. Therefore, the objective of this study was to compare soil physicochemical properties in fields with physical conservation structures not combined with biological measures (fanya juu without biological measures), physical conservation structures combined with biological measures of ten years (fanya juu with biological measures) to landscape without soil and water conservation practices. The significance of the study shows the variation of soil properties due to land management changes. This will help in decision-making by providing more information about the current status of the soil under different management. Moreover, it is helpful for the development of a soil management plan framework to maintain its sustainability and health in the study area.

## 2. Materials and Methods

### 2.1. Description of the Study Area

**Figure 1:**
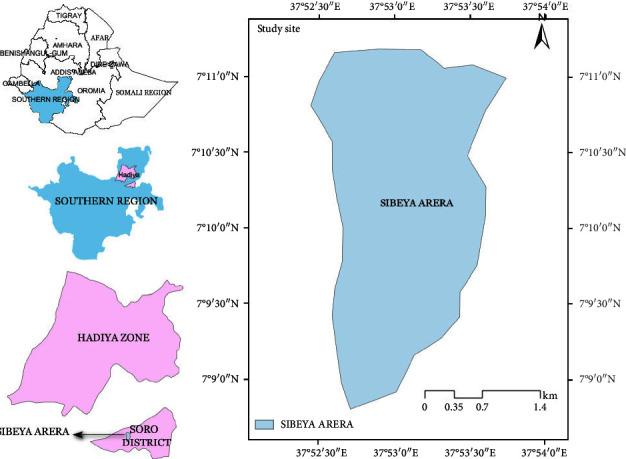
Location map of the study area.

Soro is one of the administrative districts of Hadiyya zone which is located in south central Ethiopia. It is situated approximately 272 km southwest of Addis Ababa and in a close proximity to the Gimicho town. Sibiya Arera is geographically located in 7° 9′ 0″–7° 11′ 0″ N latitude and 37° 52′ 30″–37° 54′ 0″ E longitude (Figure 1).

**Figure 2:**
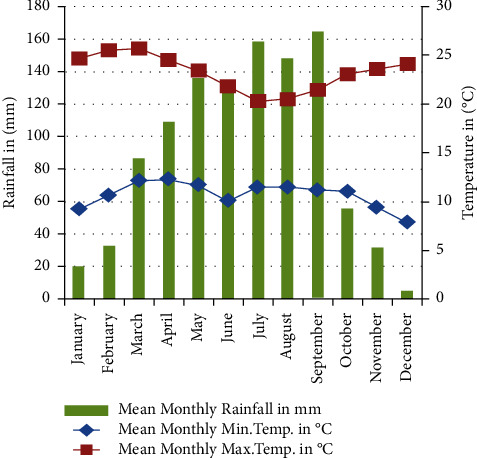
Mean monthly rainfall and temperature of the study area.

Rainfall distribution in the study area is bimodal, characterized by heavy rainy season from June to September, and light rainy season from March to May. The annual long term average rainfall is 1,107 mm and peak rainfall in September. The long term average annual temperature is 17.2°C [10]. The mean monthly temperature ranges from 15.98°C in December to 18.91°C in March (Figure 2). These favorable climatic conditions and high population have made the district to be one of the intensively cultivated areas in the south central highlands of Ethiopia. Rain-fed agriculture is the only source of livelihood for the majority of population. It is characterized by a smallholder mixed crop-livestock production.

Soil is a good indicator of the influence of soil parent material and the spatial variability in the degree of weathering, geological, and other factors are responsible for soil formation and development [11]. The dominant soil type of the study area is Nitisols that cover extensive areas of agricultural fields are highly suitable for crop production. The local geology is characterized by volcanic basalt flows and Cenozoic pyroclastic fall deposits [10].

The major land use/land cover types in the district include cultivated land, grazing land, forest land, and built-up areas. Cultivated land is the dominant land use type with 50,454 hectares (73.3% of the total area). At the present time, the local community has been implementing different practices to protect the adverse effect of erosion on their farmland and to improve soil fertility. Sibiya Arera is one of the areas with better implementation of soil and water conservation practices. Model farmers adopted biological and physical conservation practices, however, there is still land without conservation technologies which owned by reluctant farmers showing no willingness to implement soil and water conservation measures.

The farming system of the study area is mainly subsistence farming based on mixed crop-livestock production. Major crops grown in the area include wheat (*Triticum aestivum* L.), maize (*Zea mays* L.), barley (*Hordeum vulgare* L.), sorghum (*Sorghum bicolor* (L.) Moench), and teff (*Eragrostis tef* (Zucc.) Trotter). All farmers of the area have been practicing rain-fed agriculture based on continuous cultivation. Previously, diammonium phosphate (DAP) and urea were the main fertilizer types used by a large number of people. However, currently, farmers in the study area have started to use blended fertilizers such as nitrogen, phosphorus, sulfur (NPS), and nitrogen, phosphorus, sulfur, and boron (NPSB).

Arable lands are composed of the landscape without conservation practice, physical soil and water conservation structures (fanya juu), and physical soil and water conservation structures combined with biological practices (fanya juu stabilized with desho grass). Soil and water conservation practices are mechanisms used to reduce erosion and associated nutrient loss, reducing the risk of production; however, are not constructed in some agricultural lands in the study area. As a result soil erosion is major deterioration processes which lead to soil degradation and declining agricultural productivity in nonconserved agricultural land. Fanya juu structures integrated with biological practices are permanent features made of earth, designed to protect the soil from uncontrolled runoff and erosion and retain water where needed. It seeks to increase the amount of water seeping into the soil, reducing the speed and amount of water running off. Erosion is prevented by keeping enough vegetation cover on embankment to protect the soil surface and binds the soil together and maintains soil structure.

### 2.2. Design of Experimental Plots and Soil Sampling

Depending on the information obtained from the reconnaissance survey, in the study area, landscape with three different soil management practices were used as treatments to study its effects on soil properties. There are distinct differences in soil management practices among farmers in the study area. These management practices include landscape without conservation practice (control field or treatment i), physical soil and water conservation structures (fanya juu not supported with desho grass) (treatment ii), and physical soil and water conservation structures combined with biological practices or (fanya juu supported with desho grass) (treatment iii) were selected for soil data collection. Based on the landscape positions, the farm land of the study watershed was categorized into three landscape positions, such as upper landscape position (>30%), middle landscape position (15–30%), and lower landscape position (3–15%). Experimental design and arrangements were accomplished using a transect line [12]. Soil samples were collected from line transects which were laid along the contour. In each landscape position, two lines transect were laid at a distance of 50 m between them. On each line, transect five sampling points were laid at a distance of 25 m from each point. To avoid the border effect, the first and the last lines transect were laid at a distance of 5 m from the edges. We used area-based types of topographic/geographic unit sampling: Area-based soil sampling means that more than one soil sample is collected and composited from each topographic zone (landscape position). Each landscape (treatment) had ten replications. Thus, a total of 30 samples (3 treatments × 10 replications) were collected by using auger from a depth of 0–30 cm. Soil samples were collected during January 2020 to February 2020 after the crop harvest. Moreover, undisturbed soil samples were also collected separately using core samples from each land management type for the determination of soil bulk density. Disturbed soil samples placed in polythene bags and undisturbed soil samples in a steel core sampler were well labeled as described by the Soil Survey Field and Laboratory Method Manual [13] and then taken for subsequent laboratory test.

Prior to laboratory analysis, the soil samples were air dried, crushed, and passed through 2 mm sieve. Analyses of the soil samples for particle distribution, bulk density (BD), soil pH, organic carbon (OC), total nitrogen (TN), available phosphorus (AP), cation exchange capacity (CEC), and exchangeable bases were conducted at the soil fertility laboratory of the Agricultural Bureau of Southern Nations Nationalities and People's Region following standard laboratory procedures.

The soil particle size distribution was determined by a hydrometer method outlined by the simplified procedure of reference [14]. Soil textural names were determined following the textural triangle of USDA system [15]. Bulk density (BD) was estimated from undisturbed soil samples collected using a steel core sampler [16]. Soil pH (H_2_O) was measured by using a pH meter in a 1 : 2.5 soil : water [17]. The content of soil organic carbon (%) was decided by the method proposed by Walkley et al. [18]. After laboratory report, SOC content was changed to SOM content using conversion factor of 1.724 adopted from references [19, 20]. The total nitrogen was estimated by Kjeldahl methods [21]. Available phosphorus was decided by extraction from the soil using sodium carbonate at pH equals 8.5 [22]. The CEC was determined at soil pH 7 after displacement by using 1 N ammonium acetate method in which it was thereafter, estimated titrimetrically by distillation of ammonium that was displaced by sodium [23]. Exchangeable bases were determined after leaching the soils with ammonium acetate [24].

### 2.3. Statistical Analysis

ANOVA was applied to analyze the difference in mean values of soil parameters between the farm fields with physical soil and water conservation structures, physical soil and water conservation structures combined with biological measures and without conservation practices. Treatment mean comparison was determined using the least significant difference (LSD) at 0.05 level of significance [25]. Statistical Package for Social Sciences (SPSS) software, version 26.0 was used for the analysis of the data.

## 3. Results and Discussion

### 3.1. Particle-Size Distribution

The result showed that the sand content was significantly affected by soil conservation practices. Sand content of the soil had shown substantial variation with conservation practices (*P* ≤ 0.001). However, there was no significant variation in mean values of sand between fields of physical conservation structures and physical conservation structures combined with biological measures. The highest and lowest mean value (40.20 and 30.80) of sand content of the soil was recorded under the field without conservation practices and physical conservation structures combined with biological measures, respectively (Table 1). Sand percentage decreased as one go from the land without conservation practices to the land with conservation structures combined with biological measures. This difference is attributed by the variability in the problem caused by erosion. Moreover, least significant difference (LSD) test revealed that land without conservation practice showed significantly higher sand content than the rest land management types.

The result also showed that the clay content was significantly affected by soil conservation practices. Clay had shown substantial variation with conservation practices (*P* ≤ 0.001). However, there was no significant variation (*P* ≤ 0.05) in mean values of clay between fields of physical conservation structures and physical conservation structures combined with biological measures. The lowest and highest mean value (27.40 and 40.50) of clay content of the soil was recorded under the field without conservation practices and physical conservation structures combined with biological measures, respectively (Table 1). Clay percentage increased as one go from the land without conservation practices to the land with conservation structures combined with biological measures. This difference is attributed by the variability in the problem caused by erosion. Moreover, least significant difference (LSD) test revealed that land without conservation practice showed significantly lower clay content than the rest land management types.

The result showed that the silt content was significantly affected by soil conservation practices. Silt content of the soil had shown substantial variation with conservation practices (*P* ≤ 0.001). However, there was no significant variation (*P* ≤ 0.05) in mean values of silt between fields of physical conservation structures and physical conservation structures combined with biological measures. The lowest and highest mean value (28.6 and 32) of silt content of the soil was recorded under the fields without conservation practices and physical conservation practice supported by biological measures, respectively (Table 1). The mean value of silt increased as one goes from the land without conservation practices to the land with physical conservation practices supported with biological measures. This difference is attributed by the variability in the problem caused by erosion. Moreover, least significant difference (LSD) test revealed that land without conservation practice showed significantly lower silt content than the rest land management types.

### 3.2. Bulk Density (BD)

The data obtained from the laboratory were subjected to one-way analysis of variance (ANOVA) to check whether significant difference exists among different soil management practices. The result showed that the bulk density was significantly affected by soil conservation practices. Bulk density of the soil had shown substantial variation with conservation practices (*P* ≤ 0.001). However, there was no significant variation (*P* ≤ 0.05) in mean values of bulk density between fields of physical conservation structures and physical conservation practices combined with biological measures. The highest and lowest mean value (1.36 g/cm^3^ and 1.17 g/cm^3^) of soil bulk density was recorded under the field without conservation practices and physical conservation structures combined with biological measures, respectively (Table 1). The considerable increase in bulk density of soils in farm fields without conservation practice can be attributed by small amount of organic matter content available because crop residues could be used for thatch and other purposes after crop harvest. Moreover, such differences could probably be due to the influence of livestock grazing, since after harvest this farm is open for grazing. Relatively the lower bulk density in well conserved soil is obviously due to the available residue and planted grasses on conservation structures to stabilize the bunds and due to the presence of zero (restricted) grazing practice. The results obtained from this study are also in line with Hillel [26] who stated that soils of higher bulk densities are highly compacted. Least significant difference (LSD) test also revealed that farm field without conservation practice had significantly higher bulk density than the rest land management types.

### 3.3. pH (H_2_O), Soil Organic Carbon, Total Nitrogen, and Available Phosphorus

The results of the study showed significant variation of soil pH (H_2_O) with conservation practices. The pH (H_2_O) of the soil had shown substantial variation with soil and water conservation practices (*P* ≤ 0.001). The mean values (6.10) and (5.69) soil pH (H_2_O) was recorded under the farm field of conservation structure combined with biological measures and farm field without conservation practices, respectively (Table 2). The mean value of soil pH (H_2_O) is higher in the conserved land than in the nonconserved land (Table 2). However, there was no significant variation (*P* ≤ 0.05) in mean values of pH (H_2_O) between fields of physical soil and water conservation structures and physical conservation structures combined with biological measures. According to the rating proposed by Warren et al. [27], the soil pH is moderately acidic and slightly acidic in farm field without conservation practice and with conservation practices, respectively. Slightly acidic soil pH in conserved land might be associated with the decrease of the loss of soil organic matter and exchangeable bases through soil erosion and thereby increase pH of the soil. On the other hand, the reduction in pH of the soil of nonconserved farm field could be due to continuous removal of basic cations by severe erosion from the exposed surfaces of nonconserved land. This result is in agreement with findings of different researchers who observed higher pH value from the conserved farm field as compared to nonconserved farm fields [28, 29].

Soil conservation practices influenced soil organic carbon (SOC) of farm fields. Soil organic carbon (SOC) content showed significant variation with conservation practices (*P* ≤ 0.001). The mean value of SOC ranges between 1.40 and 2.37% in which physical conservation structures combined with biological measures had the highest mean value and the lowest mean value was obtained on nonconserved farm field (Table 2). This might show that soil conservation practices have played a positive role in improving the content of soil organic carbon (SOC). This finding is in accordance with Tanto et al. [30], who reported that soil organic carbon (SOC) content of cultivated land without conservation practices, was significantly lower than that of cultivated land with conservation practices.

Total nitrogen content of soils showed significant variation with conservation practices (*P* ≤ 0.001). The mean values of total nitrogen (TN) decreased with the change in conservation practices from physical conservation practices combined with biological measures (0.19%) to land without conservation practices (0.12%), following the reduction in soil organic carbon (SOC) content. According to the rating proposed by Hazelton et al. [31], total nitrogen content of the soil is medium (sufficient) and low (deficient) in farm field with conservation and without conservation practices, respectively. Generally, the result of this study is in agreement with findings of Selassie et al. [32] who reported that physical soil and water conservation practices supplemented with biological conservation measures had a positive effect in improving the content of total nitrogen (TN) of the soil.

Difference in land management practices significantly affected the available phosphorus (AP) of the soil. The result of ANOVA revealed significant difference between land management types (*P* ≤ 0.001). The lowest content of available phosphorus (8.00 ppm) was recorded in the nonconserved farmlands. Relatively higher mean value of available phosphorous was observed in farmlands of conservation structures supported by biological measures having a mean value of (11.30 ppm) (Table 2). According to the rating proposed by FAO, (2006), the content of available phosphorus of the soil is medium (optimum) and low (deficient) in farm field with conservation practices and without conservation practices, respectively. Low available phosphorus content of farm field without conservation practices might be due to the washing away of basic cations by the action of water erosion that result in soil acidity (low pH) and low organic matter. The application of lime and phosphorus in farm field without conservation practices by means of fertilizer can increase availability of phosphorus.

### 3.4. Cation Exchange Capacity and Exchangeable Bases

Difference in land management practices significantly affected the cation exchange capacity (CEC) of the soil. The result of ANOVA revealed significant difference between land management types (*P* ≤ 0.001). The lowest content of CEC (21.30 meq/100 g of soil) was recorded in the nonconserved fields of farmlands. Relatively higher mean value of CEC was observed in the fields of farmlands with conservation practices having a mean value of (28.40 meq/100 g of soil) (Table 3). According to the rating proposed by Warren et al. [27], the content of CEC of the soil is high and medium in farm field with soil conservation practices integrated with biological measures and without soil conservation practices, respectively. Low CEC content of farm field without conservation practices might be due to the wearing a way of basic cations by the action of severe erosion.

The result of analysis indicated that the mean values of exchangeable bases (Na, K, Ca, and Mg) were significantly varied (*P* ≤ 0.001). The mean relative abundance of basic cations in the exchange complex for all the land management categories in the study samples were in the order of (Ca > Mg > K > Na) (Table 3). This indicates that calcium was a distinguished dominant exchangeable base and the concentration of sodium had the smallest component on the exchange complex. High mean values of exchangeable bases (Na, K, Ca, and Mg) were recorded in conserved land. However, lower exchangeable cations (Na, K, Ca, and Mg) were found in nonconserved land as compared to conserved land. This is because of washing away of top soil by erosion result in reduction of exchangeable bases. The results of this study is failed to be in accordance with the findings of Amare et al. [33], who reported a nonsignificant variation in exchangeable bases between land with conservation and without conservation measures.

## 4. Conclusion

In the study area, different soil and water conservation (fanya juu integrated with desho grass and fanya juu only) measures were implemented by the community participation to minimize soil erosion and related problems. Besides, there is landscape without conservation practice as control field. This study was conducted to analyze the effects of soil and water conservation (fanya juu with desho grass, fanya juu only) measures on the selected soil physicochemical properties at Sibiya Arera. The results of this study showed that land management types significantly affected selected soil properties. The soil without conservation measure had resulted in significant reduction of soil nutrients, while it increased bulk density of the soil. For most parameters evaluated, the most favorable soil properties were found in soils of landscape with conservation practices (fanya juu integrated with desho grass) followed by fields with soil and water conservation (fanya juu only), while the least favorable soil properties were found in fields without conservation practices. This finding suggests the need for physical and biological conservation measures, particularly in the cultivated land, to minimize damage of erosion and improve the soil property. This would be planned and implemented based on the approach of integrated watershed management. Therefore, the study might have policy implications about how soil quality could be maintained with proper design and implementation of physical and biological soil and water conservation practices to improve the livelihood and ensure food security of the rural farming community.

## Figures and Tables

**Table 1:** Effects of land management on soil physical properties (mean ± standard deviations).

Land management types	Sand (%)	Clay (%)	Silt (%)	BD (g/cm^−3^)
Fanya juu not supported with desho grass	32.6 ± 2.413^a^	38.7 ± 3.773^a^	28.6 ± 2.119^a^	1.18 ± 0.000^a^
Fanya juu supported with desho grass	30.80 ± 1.936^a^	40.50 ± 2.455^a^	28.70 ± 1.537^a^	1.17 ± 0.000^a^
Landscape without conservation practice (control field)	40.2 ± 3.521^b^	27.4 ± 2.989^b^	32 ± 1.491^b^	1.36 ± 0.000^b^
Mean	34.43 ± 4.890	35.53 ± 6.606	29.77 ± 2.329	1.24 ± 0.000
*F*	34.362	52.974	12.251	32.439
Sig	^*∗∗∗*^	^*∗∗∗*^	^*∗∗∗*^	^*∗∗∗*^

Means within a column followed by same letters in superscripts are not significantly different from each other at *P* ≤ 0.05; ^*∗∗∗*^*P* ≤ 0.001.

**Table 2:** Effects of land management on selected soil properties (mean ± standard deviations).

Land management types	pH (H_2_O)	SOC (%)	TN (%)	AP (mg/kg)
Fanya juu not supported with desho grass	6.09 ± 0.000^a^	2.37 ± 0.483^a^	0.19 ± 0.000^a^	11.39 ± 2.547^a^
Fanya juu supported with desho grass	6.1 ± 0.000^a^	2.4 ± 0.441^a^	0.20 ± 0.000^a^	11.56 ± 2.833^a^
Landscape without conservation practice (control field)	5.69 ± 0.000^a^	1.40 ± 0.483^b^	0.12 ± 0.000^b^	8.14 ± 0.667^b^
Mean	5.96 ± 0.000	2.06 ± 0.000	0.17 ± 0.000	10.36 ± 2.200
*F*	0.255	14.286	0.151	17.807
Sig	^*∗∗∗*^	^*∗∗∗*^	^*∗∗∗*^	^*∗∗∗*^

Means within a column followed by same letters in superscripts are not significantly different from each other at *P* ≤ 0.05; ^*∗∗∗*^*P* ≤ 0.001.

**Table 3:** Effects of land management on selected soil properties (mean ± standard deviations).

Land management types	CEC (meq/100 gm soil)	Na (cmol/kg)	K (cmol/kg)	Ca (cmol/kg)	Mg (cmol/kg)
Fanya juu not supported with desho grass	28.4 ± 2.547^a^	0.23 ± 0.000^a^	0.18 ± 0.422^a^	14.46 ± 1.567^a^	6.18 ± 1.563^a^
Fanya juu supported with desho grass	28.8 ± 2.833^a^	0.24 ± 0.000^a^	1.67 ± 0.441^a^	14.47 ± 1.424^a^	6.2 ± 1.616^a^
Landscape without conservation practice (control field)	21.31 ± 0.160^b^	0.13 ± 0.000^b^	0.8 ± 0.000^b^	10.9 ± 0.738^b^	3.77 ± 0.483^b^
Mean	26.17 ± 4.161	0.2 ± 0.000	1.38 ± 0.507	13.28 ± 2.086	5.39 ± 1.675
*F*	32.901	0.000	18.00	21.189	10.328
Sig	^*∗∗∗*^	^*∗∗∗*^	^*∗∗∗*^	^*∗∗∗*^	^*∗∗∗*^

Means within a column followed by same letters in superscripts are not significantly different from each other at *P* ≤ 0.05; ^*∗∗∗*^*P* ≤ 0.001.

## Data Availability

The data used to support the findings of this study are available upon reasonable request from the corresponding author.
